# Immune responses against SARS-CoV-2 variants after two and three doses of vaccine in B-cell malignancies: UK PROSECO study

**DOI:** 10.1038/s43018-022-00364-3

**Published:** 2022-03-24

**Authors:** Sean H. Lim, Beth Stuart, Debora Joseph-Pietras, Marina Johnson, Nicola Campbell, Adam Kelly, Danielle Jeffrey, Anna H. Turaj, Kate Rolfvondenbaumen, Celine Galloway, Thomas Wynn, Adam R. Coleman, Benjamin Ward, Karen Long, Helen Coleman, Carina Mundy, Andrew T. Bates, Diana Ayres, Robert Lown, Janlyn Falconer, Oliver Brake, James Batchelor, Victoria Willimott, Anna Bowzyk Al-Naeeb, Lisa Robinson, Ann O’Callaghan, Graham P. Collins, Tobias Menne, Saul N. Faust, Christopher P. Fox, Matthew Ahearne, Peter W. M. Johnson, Andrew J. Davies, David Goldblatt

**Affiliations:** 1 Centre for Cancer Immunology, University of Southampton, Southampton, UK; 2 Cancer Research UK Research Centre, University of Southampton, Southampton, UK; 3 University Hospital Southampton NHS Foundation Trust, Southampton, UK; 4 Cancer Research UK Southampton Clinical Trials Unit, University of Southampton and University Hospital Southampton NHS Foundation Trust, Southampton, UK; 5 NIHR/Cancer Research UK Southampton Experimental Cancer Medicine Centre, WISH Laboratory, Southampton General Hospital, Southampton, UK; 6 Great Ormond Street Institute of Child Health Biomedical Research Centre, University College London, London, UK; 7 University of Southampton Clinical Informatics Research Unit, Southampton General Hospital, Southampton, UK; 8 Norfolk and Norwich University Hospitals NHS Foundation Trust, Norwich, UK; 9 Department of Oncology, Bedford Hospital, Bedford, UK; 10 Department of Haematology, County Hospital Hereford, Hereford, UK; 11 Portsmouth Hospitals NHS Trust, Portsmouth, UK; 12 Department of Clinical Haematology, Oxford University Hospitals NHS Foundation Trust, Oxford, UK; 13 Department of Haematology, Newcastle upon Tyne Hospitals NHS Foundation Trust, Newcastle Upon Tyne, UK; 14 Faculty of Medicine and Institute for Life Sciences, University of Southampton, Southampton, UK; 15 NIHR Southampton Clinical Research Facility and NIHR Southampton Biomedical Research Centre, University Hospital Southampton NHS Foundation Trust, Southampton, UK; 16 Nottingham University Hospitals NHS Trust, Nottingham, UK; 17 University Hospitals of Leicester NHS Trust, Leicester, UK

**Keywords:** Lymphoma, Cancer, Vaccines, SARS-CoV-2

## Abstract

Patients with hematological malignancies are at increased risk of severe COVID-19 outcomes due to compromised immune responses, but the insights of these studies have been compromised due to intrinsic limitations in study design. Here we present the PROSECO prospective observational study (NCT04858568) on 457 patients with lymphoma that received two or three COVID-19 vaccine doses. We show undetectable humoral responses following two vaccine doses in 52% of patients undergoing active anticancer treatment. Moreover, 60% of patients on anti-CD20 therapy had undetectable antibodies following full vaccination within 12 months of receiving their anticancer therapy. However, 70% of individuals with indolent B-cell lymphoma displayed improved antibody responses following booster vaccination. Notably, 63% of all patients displayed antigen-specific T-cell responses, which increased after a third dose irrespective of their cancer treatment status. Our results emphasize the urgency of careful monitoring of COVID-19-specific immune responses to guide vaccination schemes in these vulnerable populations.

## Abstract (web summary)

Lim and colleagues present the UK PROSECO study where they assess humoral and cellular immune responses to one, two and three doses of vaccination against SARS-CoV-2 in patients with B-cell malignancies in a prospective observational study.

## Main

Individuals with cancer have increased morbidity and mortality from COVID-19 infection^1,2^. This is most apparent in patients with hematological malignancies, who have a reported odds ratio of 1.57 to 3.3 in developing severe COVID-19, compared to patients with solid tumors^1,2^. SARS-CoV-2 vaccination has been shown to be highly efficacious in preventing symptomatic COVID-19 disease in healthy individuals^3,4^. Recent data indicate that the presence of both binding and neutralizing antibodies is highly predictive of protection against symptomatic disease, although a threshold correlating with protection is yet to be determined^5,6^. A number of observational studies have reported that while the majority of patients with solid malignancies develop anti-spike (S) IgG antibodies to vaccination, a substantial number of patients with hematological malignancies, in particular those with lymphoid malignancies, do not^7–15^. In patients with lymphoid malignancies, a notable proportion of those who are vaccinated while receiving or recently completed B-cell-depleting or targeted treatments such as anti-CD20 monoclonal antibodies and Bruton’s tyrosine kinase (BTK) inhibitors do not have detectable antibodies. In our initial interim analysis, we also reported that a proportion of individuals with chronic, indolent B-cell malignancies have impaired serologic responses regardless of relationship to the time of treatment^7^.

Less well-explored is the role of vaccine-induced antigen-specific T cells in mediating protection, specifically a T-cell response to peptides expressing the SARS-CoV-2 S domain. Many of the SARS-CoV-2 vaccines induce demonstrable T-cell responses but due to the technical complexities of assessing a T-cell response, the majority of observational studies have not incorporated cellular responses after vaccination^7–12,14,15^.

The other important factor in evaluating the immune response elicited by the vaccine is the functional quality of the antibodies produced. Anti-S antibodies can protect against SARS-CoV-2 infection by a number of different mechanisms, which includes binding to the receptor-binding domain (RBD) within the S protein to sterically block its subsequent binding to the host ACE2 receptor and therefore viral entry into the cell^16^. Although live virus neutralization is the gold standard for assessing the presence of neutralizing antibodies against SARS-CoV-2, this is a specialist assay with limited global capacity. Instead, pseudoneutralization assays which quantify the ability of patient serum or plasma to inhibit the interaction between viral spike protein and the soluble ACE2 receptor have been shown to be a useful surrogate for functional assessment^17–19^.

To address these points, we conducted a UK multicenter prospective observational study evaluating COVID-19 vaccine responses in individuals with lymphoma (PROSECO; NCT04858568)^7^. This analysis reports serological, cellular and pseudoneutralization responses from 457 participants with lymphoma after two and three doses of either ChAdOx1 nCoV-19 (ChAdOx1) or BNT162b2 vaccines.

## Results

### Baseline characteristics

There were 457 participants with serological data. Twenty-nine participants had detectable anti-nucleocapsid (N) IgG antibodies, indicating previous SARS-CoV-2 infection and were excluded from analysis.

The baseline demographics, clinical characteristics and treatment details are described in Tables 1 and 2. Participants had Hodgkin lymphoma (HL; *n* = 71), aggressive B-cell non-Hodgkin lymphoma (B-NHL; *n* = 149), indolent B-NHL (*n* = 221) and peripheral T-cell/natural killer (NK) cell lymphoma (PTCL; *n* = 16). Participants with HL were younger than the other disease groups, with a median age of 40 years compared to 63 to 67 years, reflective of the presentation age of HL. In the aggressive B-NHL cohort, diffuse large B-cell lymphoma (DLBCL) was diagnosed in 83% (124 out of 149) of participants. In the indolent B-NHL cohort, follicular lymphoma (FL) comprised 42.1% (93 out of 221) of cases, followed by 23.1% (51 out of 221) chronic lymphocytic leukemia (CLL), 9.5% (21 out of 221) mantle cell lymphoma, 9.5% (21 out of 221) marginal zone lymphoma and 7.7% (17 out of 221) lymphoplasmacytic lymphoma. The PTCL cohort was heterogeneous with angioimmunoblastic T-cell lymphoma comprising 43.8% (7 out of 16) of the cohort. In each disease group, more patients received ChAdOx1 than BNT162b2 vaccines (overall, 60.4% (276 out of 457) versus 39.4% (180 out of 457), respectively).

**Table 1:** Baseline characteristics of participants

	HL	Aggressive B-NHL	Indolent B-NHL	PTCL
**Number of cases**	71	149	221	16
**Age in years, median (IQR)**	40 (29–54)	67 (58–73)	67 (58–73)	63 (54–68)
**Sex**				
**Male,** ***n*** **(%)**	44 (62%)	83 (56%)	137 (62%)	8 (50%)
**Female, (%)**	27 (38%)	66 (44%)	84 (38%)	8 (50%)
**Vaccine type**				
**ChAdOx1,** ***n*** **(%)**	43 (61%)	95 (64%)	128 (58%)	10 (63%)
**BNT162b2,** ***n*** **(%)**	28 (39%)	54 (36%)	93 (42%)	5 (31%)
**NA**				1 (6%)
**Disease subtypes (no. of cases)**	NA	DLBCL (124)	FL (93)	EATL (2)
		Transformed FL/MZL (6)	CLL (51)	AITL (7)
		PBML (3)	MCL (21)	BIA-ALCL (1)
		PCNSL (6)	LPL (17)	ALCL (3)
		Burkitt lymphoma (4)	MZL (21)	Subcutaneous panniculitis T-cell lymphoma (1)
		High grade B-cell lymphoma (1)	Hairy cell leukemia (2)	
		Richter’s transformation (2)	NLPHL (6)	
		T-cell rich DLBCL (1)	Low grade B-NHL (6)	
		PTLD (1)	SLL (4)	
		Systemic CNS lymphoma (1)		
**Treatment group**				
**‘No treatment’**				
**None,** ***n*** **(%)**	1 (1%)	1 (1%)	54 (24%)	0 (0%)
**Previous,** ***n*** **(%)**	31 (44%)	100 (67%)	82 (37%)	11 ((69%)
**‘On treatment’,** ***n*** **(%)**	39 (55%)	48 (32%)	85 (38%)	5 (31%)
**Number of lines of treatment, median (range)**	1 (0–4)	1 (0–8)	1 (0–5)	3 (0–4)
**Previous autologous stem cell transplant**	4	15	13	4
**Previous allogeneic stem cell transplant**	2	1	1	0
**Previous CAR-T-cell therapy**	0	3	2	0
**Remission status**				
**CR/PR,** ***n*** **(%)**	52 (73%)	102 (68%)	129 (58%)	11 (69%)
**PD,** ***n*** **(%)**	0 (0%)	10 (7%)	10 (5%)	0 (0%)
**SD** ***n*** **(%)**	5 (7%)	7 (5%)	44 (20%)	3 (19%)
**Not yet assessed,** ***n*** **(%)**	14 (20%)	30 (20%)	38 (17%)	3 (19%)
**Previous COVID-19**	4	11	12	2

PCNSL, primary central nervous system lymphoma; MCL, mantle cell lymphoma; MZL, marginal zone lymphoma; EATL, enteropathy associated T-cell lymphoma; AITL, angioimmunoblastic T-cell lymphoma; BIA-ALCL, breast implant associated anaplastic large cell lymphoma; ALCL, anaplastic large cell lymphoma; NLPHL, nodular lymphocyte predominant Hodgkin lymphoma; CNS, central nervous system; CR, complete remission; PR, partial remission; PD, progressive disease; SD, stable disease; NA, not available

**Table 2:** Treatments received by participants within the ‘On Treatment’ group

Last line of treatment	HL	Aggressive B-NHL	Indolent B-NHL	PTCL
**Chemotherapy**	27	0	0	1
**Anti-CD20 alone**	0	2	27	0
**Anti-CD20 + chemotherapy**	0	36	14	0
**Anti-CD20 + chemotherapy-free agent**	0	1	6	0
**Bendamustine**	1	2	0	0
**Bendamustine + anti-CD20**	0	0	5	0
**BTK inhibitors**	0	0	17	0
**Venetoclax**	0	0	7	0
**BTK inhibitor + venetoclax**	0	0	2	0
**CAR-T-cell therapy**	0	0	0	0
**Autologous stem cell transplant**	0	4	2	0
**Allogeneic stem cell transplant**	0	0	0	0
**Others**	BV (2), BV + bendamustine (1)	CPI (1) Bispecific Ab (1)	Bispecific Ab + lenalidomide (1)	PI3K inhibitor (1) BV (1) Ciclosporin (1)

BV, brentuximab vedotin; CP, checkpoint inhibitor; PI3K, phosphatidylinositol 3-kinase

Overall, 38.7% (177/457) of participants were in the ‘on treatment’ group, which was defined as those who received their first vaccine dose within 24 weeks of completing systemic anti-lymphoma treatment or whose treatment had commenced within 4 weeks after receiving the first vaccine dose. Forty-nine percent (224 out of 457) of participants had completed their treatment more than 24 weeks before their first vaccine dose and were allocated to the ‘no treatment’ group. A further 12.3% (56 out of 457) participants had not received systemic treatment for their disease before the first vaccine dose; comprising patients with indolent B-NHL (96.4%, 54 out of 56) who were also allocated to the ‘no treatment’ group.

**Fig. 1:**
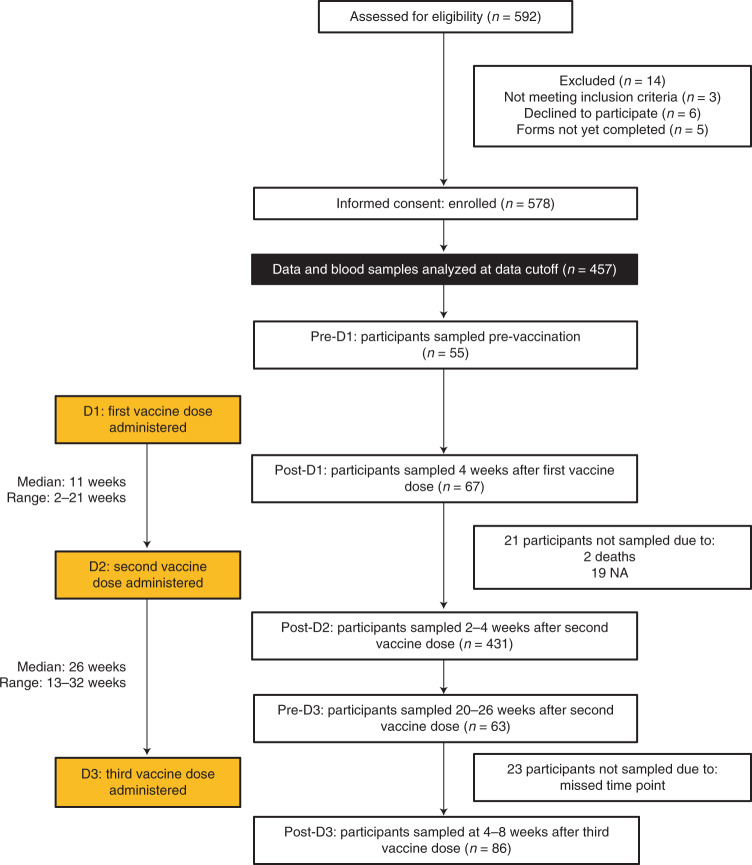
CONSORT diagram of study cohort. A total of 592 patients were assessed for eligibility and subsequently 578 were recruited into the study. At the time of data cutoff, blood samples were processed from 457 participants. The vaccination schedule and numbers of participants sampled at the following time points are shown: pre-vaccination (pre-D1), 4 weeks after first dose (post-D1), 2–4 weeks after second dose (post-D2), 20–26 weeks after second dose (pre-D3) and 4–8 weeks after third dose (post-D3).

Anti-S, anti-RBD and anti-nucleocapsid (N) IgG concentrations were measured in 55, 67 and 430 participants before vaccination (‘pre-D1’), 4 weeks after first dose (‘post-D1’) and 2–4 weeks after second dose of vaccine (‘post-D2’), respectively (Fig. 1). Thirty-two participants had cellular responses investigated by paired T-cell interferon (IFN)-γ ELISpot testing on peripheral blood mononuclear cells (PBMCs) collected pre-D1 and post-D2. A further 159 participants had ELISpot testing only at post-D2. Peripheral blood T, B and NK cell counts were also simultaneously performed alongside ELISpot testing. The ACE2 receptor-blocking assay was performed on 282 post-D2 samples. Serum was available from 136 healthy donor volunteers for serological analysis and a further 11 donors for ELISpot and T, B and NK analysis, respectively. Pre-D3 (20–26 weeks after second dose) and post-D3 (4–8 weeks after third dose) sampling is described later.

### Treatment impairs antibody but not cellular responses

**Fig. 2:**
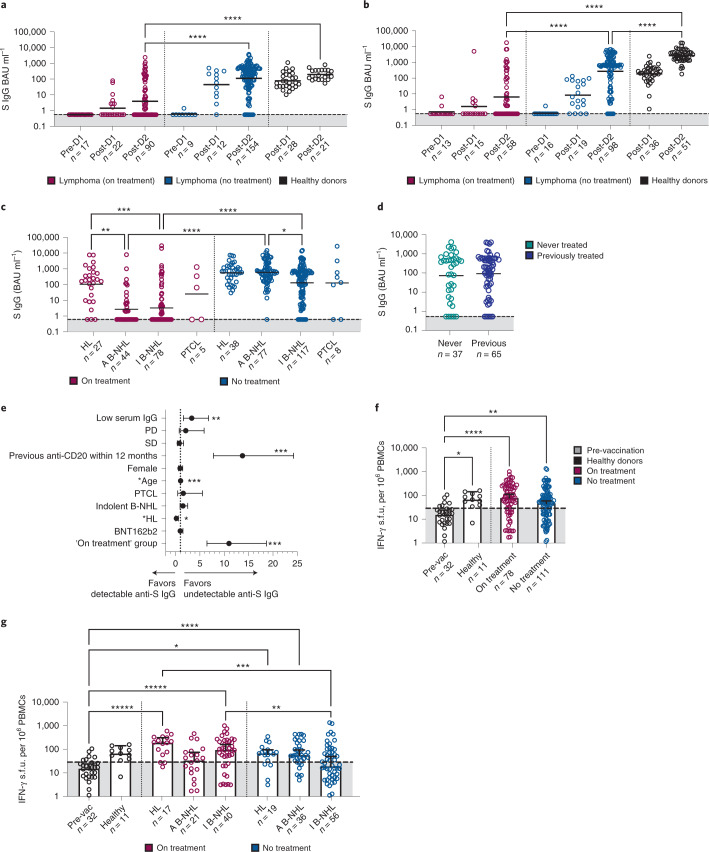
Antibody responses but not cellular responses to SARS-CoV-2 vaccination are impaired by systemic therapy. **a**, Participants with lymphoma vaccinated with ChAdOx1 vaccine while on systemic treatment or within 24 weeks of treatment completion (designated ‘on treatment’) had reduced antibody levels compared to participants with previous or untreated lymphoma (designated ‘no treatment’) and healthy controls (designated ‘healthy donors’). The dashed line and shaded region indicate undetectable antibody. GMC is shown; two-sided Kruskal–Wallis test, Dunn’s test for multiple comparisons, ****P < 0.0001. **b**, As in **a** but with BNT162b2 where the same phenomenon is observed. **c**, Reduced anti-S IgG antibody levels in HL, aggressive B-NHL (A B-NHL) and indolent B-NHL (I B-NHL) participants in the ‘on treatment’ group and in I B-NHL participants in the ‘no treatment’ group despite two vaccine doses. GMC is shown; two-sided Kruskal–Wallis test, Dunn’s test for multiple comparisons, *P = 0.0288, **P = 0.0008, ***P = 0.0004, ****P < 0.0001. **d**, Anti-S levels in participants with I B-NHL are similar between those who had received previous antisystemic therapy compared to treatment-naive. GMC is shown; two-sided Kruskal–Wallis test. *P* <0.05 was considered significant. **e**, Univariable logistic regression showing that low serum IgG, anti-CD20 exposure 12 months preceding the first vaccine dose; ‘on treatment’ group and older age (represented by ‘Age’ as a continuous variable) were associated with an increased OR of having undetectable anti-S IgG. Participants with HL favored detectable anti-S IgG. *n* = 428 participants. Error bars represent 95% CI. *P = 0.005, **P = 0.001, ***P < 0.001. **f**, Similar IFN-γ cellular responses in participants with lymphoma between ‘on’ and ‘no treatment’ groups. The dashed line and shaded region indicate a negative IFN-γ response. Median and 95% CI are shown; two-sided Kruskal–Wallis test, Dunn’s test for multiple comparisons, *P = 0.0132, **P = 0.0023, ****P < 0.0001. **g**, Reduced IFN-γ cellular responses were preserved in participants with lymphoma except for those with I B-NHL in the ‘no treatment’ group. The dashed line and shaded region indicate a negative IFN-γ response. Median and 95% CI are shown; two-sided Kruskal–Wallis test, Dunn’s test for multiple comparisons, *P = 0.0356, **P = 0.0262, ***P = 0.0093, ****P = 0.0010, *****P < 0.0001. Source data

The lymphoma participants were analyzed based on vaccine received: ChAdOx1 or BNT162b2 vaccines (Fig. 2a,b). The proportion of participants who had undetectable antibodies after two doses of each vaccine was similar in the ‘no treatment’ group (ChAdOx1 (9.1%, 14 out of 154) versus BNT162b2 (8.2%, 8 out of 98)). While participants who had received two doses of BNT162b2 achieved a 2.4-fold higher antibody level than ChAdOx1 recipients, this was not statistically significant (geometric mean concentration (GMC) 270.8 (95% CI 156.9, 467.6) versus 111.5 (78.35, 158.7) binding antibody units (BAU) ml^−1^). In healthy donors, BNT162b2 induced higher anti-S IgG antibody levels than ChAdOx1, as observed in our earlier analysis^7^. The GMC of healthy donors vaccinated with two doses of BNT162b2 was 11-fold higher than ChAdOx1 (2,667 versus 196 BAU ml^−1^).

Participants in the ‘on treatment’ group had impaired antibody responses compared to ‘no treatment’ regardless of vaccine type. Overall, 52.3% (78 out of 149) of participants ‘on treatment’ had undetectable antibodies after two doses of vaccine compared to 8.7% (22 out of 252) in the ‘no treatment’ group. Given that there was no statistical difference in antibody levels or the proportion of undetectable antibodies between the two vaccines in participants with lymphoma, ChAdOx1 and BNT162b2 groups were merged for subsequent analysis.

Within the ‘on treatment’ group, fewer participants with HL had undetectable antibodies compared to aggressive and indolent B-NHL (11.1%, (3 out of 27) versus 56.8% (25 out of 44) versus 62.7% (47 out of 78), respectively) (Fig. 2c). The sample size of the PTCL group was too small to comment on treatment effect. Overall 30.7% (4 out of 13) of participants with PTCL had no detectable antibodies. Consistent with Fig. 2a, more participants in the ‘no treatment’ group had detectable antibodies. Specifically, 100% (38 out of 38) of HL and 97.7% (86 out of 88) of aggressive B-NHL cases had detectable antibodies and 90.6% (106 out of 117) of participants with indolent B-NHL. The anti-S level was similar for HL and aggressive B-NHL but reduced for indolent B-NHL (GMC 502.9 versus 539.1 versus 116.5 BAU ml^−1^). In those with indolent B-NHL, there was no difference in the antibody level between participants who had never received systemic therapy for their disease versus those who were previously treated, suggesting that participants with indolent B-NHL may have additional treatment unrelated disease-intrinsic immune dysfunction that impairs antibody levels (Fig. 2d).

A univariable analysis showed that participants in the ‘on treatment’ group (odds ratio (OR) 10.96), anti-CD20 administration in the last 12 months (OR 13.72), low serum IgG level (OR 3.32) and increasing age (OR 1.04) were associated with an increased risk of having no detectable anti-S IgG (Fig. 2e and Supplementary Table 1). On multivariable analysis, ‘on treatment’ group (OR 7.22) and anti-CD20 administration in the last 12 months (OR 5.60) continued to be statistically significant factors (Supplementary Table 2). Serum IgG results were only available for 210 participants, so it was not included for multivariable analysis to avoid reducing the statistical power of the analysis.

The antigen-specific T-cell response in the lymphoma group, as analyzed by IFN-γ ELISpot assay using PBMCs, showed no statistically significant difference between ‘on’ and ‘no treatment’ groups (median 76.2 IFN-γ spot-forming units (s.f.u.) per 10^6^ PBMCs (95% CI 50.8, 110.0) versus 45.10 (27.97, 57.13)) (Fig. 2f). However, there was a variation in cellular response within the disease groups (Fig. 2g). In HL, the proportion of positive cellular responses was equivalent between ‘on’ and ‘no’ treatment groups (75% (12 out of 16) versus 73.9% (17 out of 23)). In contrast, 52% (11 out of 21) of participants with aggressive B-NHL who were ‘on treatment’, had a positive cellular response compared to 76.5% (26 out of 34) of participants in the ‘no treatment’ group, although there was no statistical difference in the actual number of IFN-γ s.f.u. per 10^6^ PBMCs between the two groups. In indolent B-NHL, those in the ‘no treatment’ group had an inferior response compared to those on treatment (positive responses, 44.6% (25 out of 56) versus 72.5% (29 out of 40)) and this was reflected by a statistically significant difference in the number IFN-γ s.f.u. per 10^6^ PBMCs (‘no’ versus ‘on treatment’, median 18.47 versus 91.09).

### B-cell numbers predict antibody response post anti-CD20

**Fig. 3:**
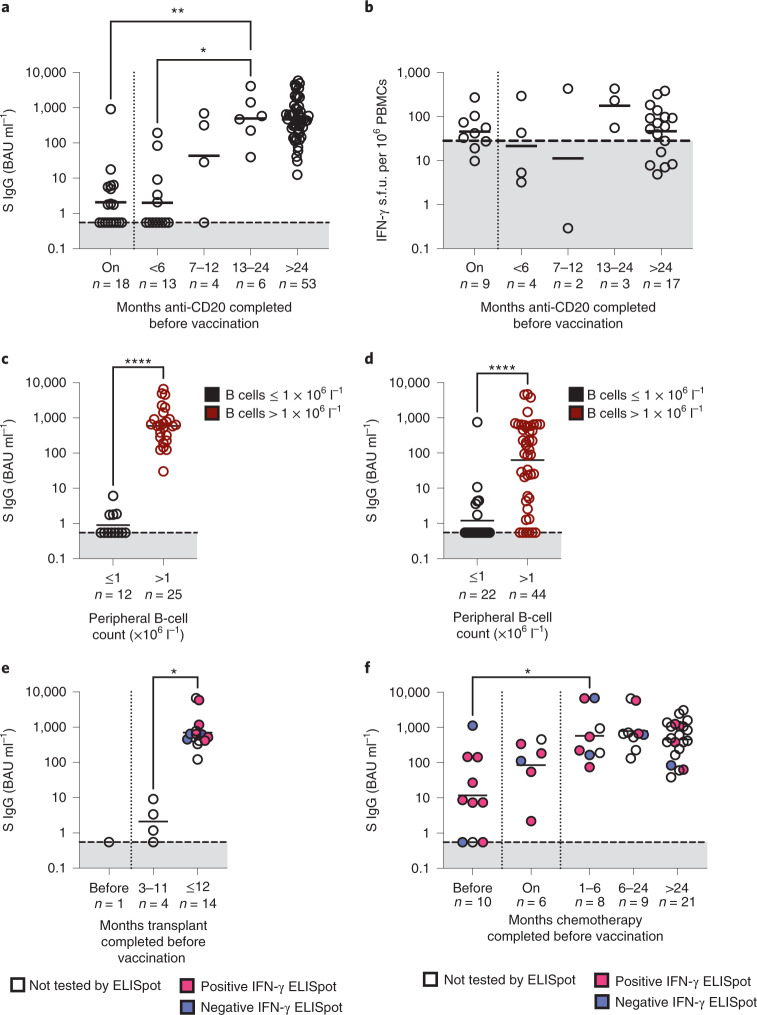
The effect of specific therapies on antibody and cellular responses. **a**, Anti-S IgG levels in participants vaccinated while receiving anti-CD20 (on) or <6, 7–12, 13–24 or >24 months of completion of treatment. The dashed line and shaded region indicate undetectable antibody. GMC is shown; two-sided Kruskal–Wallis test, Dunn’s test for multiple comparisons, *P = 0.0057, **P = 0.0060. **b**, Preservation of cellular responses in anti-CD20-treated lymphoma participants, regardless of treatment timing. All points below the dashed line represent a negative test. Medians are shown; two-sided Kruskal–Wallis test, *P* < 0.05 was considered significant. **c**, A reduced peripheral blood B-cell count is associated with impaired anti-S IgG level in aggressive B-NHL. GMC is shown; two-sided Mann–Whitney test, ****P < 0.0001. **d**, As in **c** but for indolent B-NHL; two-sided Mann–Whitney test, ****P < 0.0001. **e**, Anti-S IgG levels in participants vaccinated ≤3 months of autologous stem cell transplant have reduced anti-S. GMC is shown; two-sided Kruskal–Wallis test, Dunn’s test for multiple comparisons, *P = 0.0112. Matching antigen-specific T-cell responses are also shown. **f**, Participants with HL have reduced anti-S when vaccinated within the 3-month period before (designated ‘before’) commencing chemotherapy. GMC is shown; two-sided Kruskal–Wallis test. Dunn’s test for multiple comparisons, *P = 0.0455. Antigen-specific T-cell responses are detectable in participants regardless of timing of chemotherapy. Source data

It is recognized that anti-CD20 monoclonal antibody therapy profoundly suppresses the antibody response to SARS-CoV-2 vaccination. To ensure that the impairment in antibody response is not confounded by disease-related immune dysfunction, only participants with aggressive B-NHL were analyzed for the effect of anti-CD20 (*n* = 94) (Fig. 3a). Fifty percent (9 out of 18) and 61.5% (8 out of 13) of participants who received their first vaccine dose while on anti-CD20 or within 6 months of completion, respectively, had undetectable antibodies. Responses improved as the duration between completion of anti-CD20 before vaccination increased. One of four participants had undetectable antibodies when anti-CD20 had completed 7–12 months pre-vaccination. All participants who were vaccinated more than 12 months after anti-CD20 completion had detectable anti-S IgG (GMC 492.5 BAU ml^−1^ (95% 98.96, 2,609)).

With regard to T-cell response, a positive response was observed in 62.5% (10 out of 16) and 71.4% (15 out of 21) participants who had completed anti-CD20 less than 12 months or more before vaccination, suggesting that cellular responses are often preserved with anti-CD20 treatment (Fig. 3b).

The antibody impairment mediated by anti-CD20 therapy is very likely to be related to B-cell depletion. Therefore, we compared the antibody responses of participants with a peripheral blood B-cell count of ≤1 or >1 × 10^6^ l^−1^, within aggressive B-NHL (Fig. 3c). There was a marked and significant difference in antibody level between those with B-cell count ≤1 versus >1 (0.90 versus 590.1 BAU ml^−1^). For indolent B-NHL, a B-cell count exceeding 1 × 10^6^ cells l^−1^ was also associated with a higher antibody level but 13.6% (6 out of 44) of participants in this group still had undetectable antibodies, suggesting that other factors beyond B-cell depletion contribute to impairment of vaccine antibody response (Fig. 3d).

### Early vaccination after transplant impairs antibody response

The impact of stem cell transplantation on vaccine immune responses was examined by analysis of individuals whose last line of treatment was an autologous stem cell transplant (Fig. 3e). Here, 5 out of 19 participants who had received two doses of vaccine either 3 weeks before transplantation or within 5 months after transplantation, had a reduced antibody response compared to those vaccinated 12 months or more after transplantation (GMC 1.61 BAU ml^−1^ (95% CI 0.35, 7.31) versus 707 BAU ml^−1^ (95% CI 384.4, 1,300)). T-cell ELISpot was undertaken in eight participants who were transplanted more than 12 months ago and three out of eight participants had a negative response (Fig. 3e).

### Antibody and cellular responses are observed in HL

To assess the effect of chemotherapy on vaccine immune responses without the contribution of anti-CD20, we focused on a cohort of 54 participants with HL who had post-D2 antibody levels (Fig. 3f). Three out of ten participants who were vaccinated within 12 weeks of starting chemotherapy had undetectable antibody responses compared to one out of six participants who were vaccinated on chemotherapy. A lower antibody level was observed in the former but this was not statistically significant (GMC 11.7 BAU ml^−1^ (95% CI 1.78, 77.7) versus 85.18 BAU ml^−1^ (95% CI 11.0, 659.0)). However, those who were vaccinated before chemotherapy commencement had lower antibody levels compared to those who were vaccinated 1 month or more after treatment completion (GMC 11.7 BAU ml^−1^ (95% CI 1.78, 77.7) versus 547.5 BAU ml^−1^ (95% CI 351.5, 852.8)).

Cellular responses were also examined in 31 participants with HL. No correlation was observed between antibody and cellular responses and the proportion with positive cellular response was similar irrespective of the time of treatment (vaccination before and on chemotherapy 79% (11 out of 14) versus 76% (13 out of 17) vaccinated more than a month after chemotherapy (Fig. 3f)).

Altogether, our data suggest that cellular responses are preserved in the majority of participants on chemotherapy in HL. Antibody responses are impaired when individuals are vaccinated shortly before (3 months) or during chemotherapy compared to those who are vaccinated after treatment completion.

### No correlation between antibody and cellular responses

**Fig. 4:**
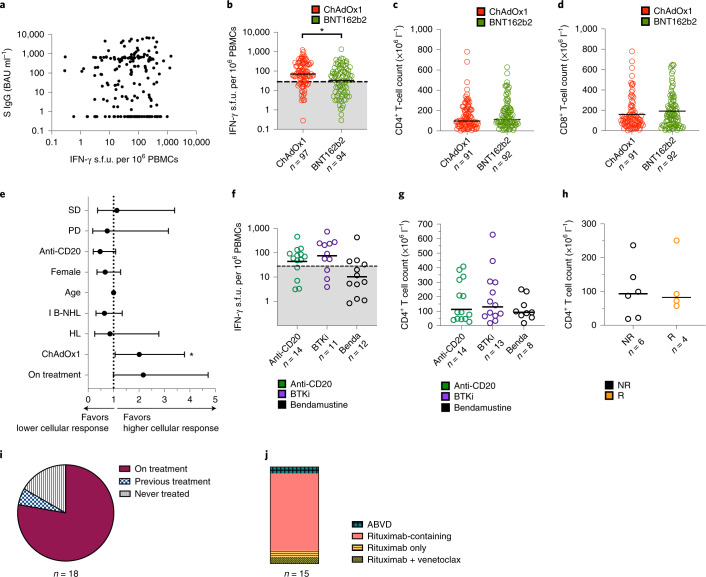
Correlates of antigen-specific T-cell responses. **a**, Lack of correlation between antigen-specific IFN-γ T-cell responses and anti-S levels. *n* = 191 participants with paired T-cell and anti-S levels. **b**, ChAdOx1 vaccination induces higher IFN-γ T-cell responses than BNT162b2. All points below the dashed line represent a negative result. Medians are shown; two-sided Mann–Whitney test, *P = 0.0005. **c**, Similar CD4^+^ T-cell counts were observed between ChAdOx1 and BNT162b2 recipients. Medians are shown; two-sided Mann–Whitney test, *P* < 0.05 was considered significant. **d**, Similar CD8^+^ T-cell counts were observed between ChAdOx1 and BNT162b2 recipients. Medians are shown; two-sided Mann–Whitney test, *P* < 0.05 was considered significant. **e**, ChAdOx1 vaccination was associated with a lower cellular response on multivariable logistic regression analysis. Error bars represent 95% confidence intervals, *n* = 191 participants, *P = 0.031. **f**, Bendamustine (Benda) treatment was associated with a trend toward reduced IFN-γ T-cell responses. All points below the dashed line represent a negative test. Medians are shown; two-sided Kruskal–Wallis test. *P* < 0.05 was considered significant. **g**, No difference in CD4^+^ T-cell counts between participants on or recently treated with anti-CD20, BTK inhibitor (BTKi) or bendamustine. Medians are shown; two-sided Kruskal–Wallis test. *P* < 0.05 was considered significant. **h**, No difference in CD4^+^ T cells was observed in bendamustine-exposed individuals who did not have an antigen-specific T-cell response (NR) compared to those who had a positive test (R); two-sided Kruskal–Wallis test. NR, non-responder; R, responder. *P* < 0.05 was considered significant. **i**, Pie chart showing that the majority of participants with both negative antibody and cellular responses were from the ‘on treatment’ group. **j**, Bar chart showing that within the double-negative ‘on treatment’ group in **i**, most participants had received combination therapy containing anti-CD20 rituximab. Source data

Predictors of cellular response to SARS-CoV-2 vaccination are poorly described compared to serological responses. Consistent with the preceding data shown here within the specific treatment groups (Figs. 2e and 3f), no correlation was observed between cellular responses and anti-S IgG levels across all 191 individuals analyzed (Fig. 4a). Despite comparable CD4^+^ and CD8^+^ T-cell counts, ChAdOx1 vaccination resulted in higher T-cell responses than BNT162b2, producing a 2.1-fold higher response (median 69.7 IFN-γ s.f.u. per 10^6^ PBMCs (95% CI 55.45, 97.50) versus 33.25 (16.68, 56.28), respectively) (Fig. 4b–d). In a multivariable analysis, vaccine type continued to be a statistically significant factor, with ChAdOx1 recipients having an increased odds ratio of a positive cellular response (OR 2.01, 95% CI 1.06, 3.79) (Fig. 4e and Supplementary Table 3). No correlation was observed between cellular response and age, sex and remission status or across disease groups.

Purine analog-like chemotherapies such as bendamustine have previously been shown to reduce peripheral blood CD4^+^ T cells beyond 3 years after completion of treatment, thereby potentially impairing cellular responses^20,21^. We compared participants with indolent B-NHL who were receiving, or had received, bendamustine-containing regimens as the last line of regimen to those treated with anti-CD20 or BTK inhibitors (Fig. 4f). Twenty-seven percent (4 out of 12) of bendamustine-treated participants had a positive response compared to 71.4% (10 out of 14) and 81.8% (9 out of 11) of anti-CD20 and BTK inhibitor-treated participants. No difference was observed in the absolute CD4^+^ T-cell counts subset counts between the three groups, or between positive and negative responders within the bendamustine cohort (Fig. 4g,h). Altogether these data suggest that unlike serological responses, systemic therapy is a poor predictor of cellular response in lymphoid malignancies.

### Factors determining a double-negative immune response

Theoretically, participants who have neither detectable antibodies nor cellular responses may be at greatest risk of a severe outcome to COVID-19 infection. In our cohort, 191 participants had paired serological and cellular response data 2–4 weeks after second vaccination. Nine percent (18 out of 191) of participants had undetectable anti-S IgG antibodies and a negative cellular response (Fig. 4i). Seventy-eight percent (14 out of 18) participants were from the ‘on treatment’ group (8 indolent B-NHL, 5 aggressive B-NHL and 1 HL). Apart from the participant with HL, all were receiving anti-CD20-containing chemoimmunotherapy (Fig. 4j). In the remaining 4 out of 18 participants from the ‘no treatment’ group, 3 had treatment-naive indolent B-NHL.

To provide perspective for these data, 10.3% (8 out of 78) of participants with indolent B-NHL, 11% (5 out of 44) with aggressive B-NHL and 3.7% (1 out of 27) with HL from the ‘on treatment’ group had no cellular or antibody response to two doses of vaccine. Seven percent (4 out of 56) of participants with indolent B-NHL in the ‘no treatment’ group also had undetectable cellular or antibody response.

### Anti-S levels correlate with virus pseudoneutralization

**Fig. 5:**
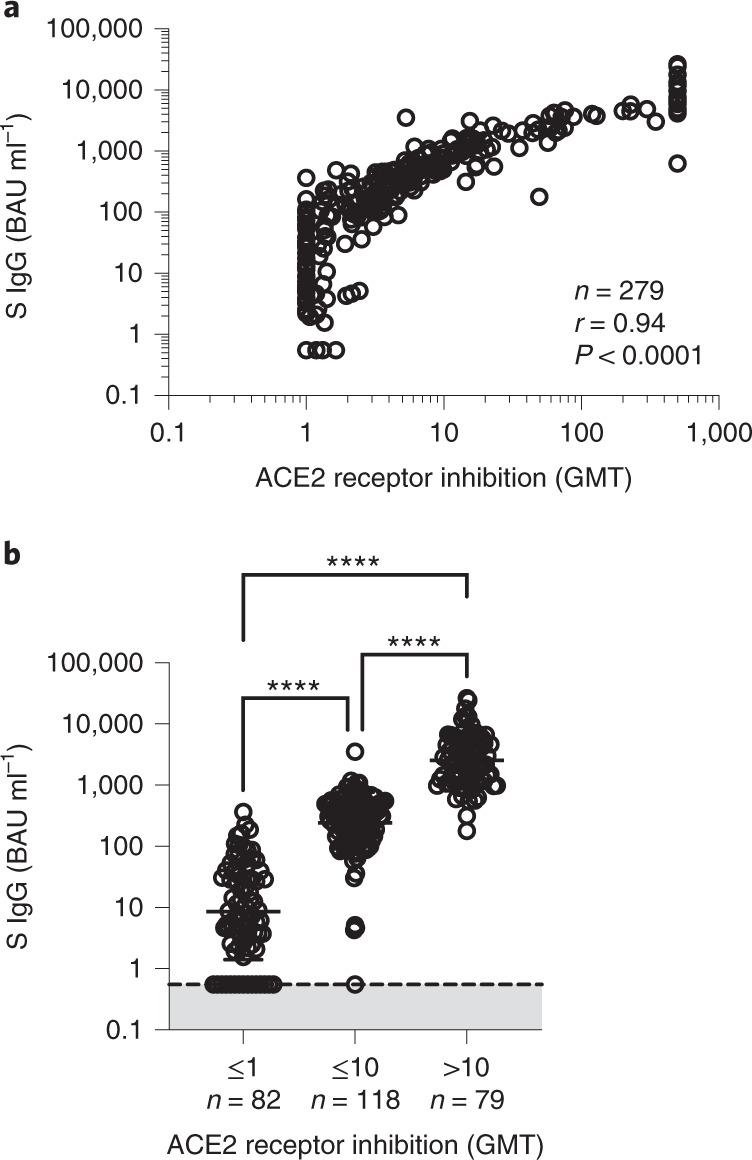
Anti-S level correlates with ACE2 receptor inhibition against wild-type SARS-CoV-2. **a**, Correlation between anti-S IgG and ACE2 receptor inhibition; two-sided Spearman test. **b**, Data from **a** but grouped according to ACE2 receptor inhibition titers; two-sided Kruskal–Wallis test, Dunn’s test for multiple comparisons, ****P < 0.0001. Source data

We also evaluated the correlation between anti-S IgG level and functional ability of the antibody to block ACE2 to wild-type spike protein using the ACE2 receptor inhibition assay (Fig. 5a,b). A good agreement was observed between anti-S IgG level and ACE2 receptor inhibition in 282 individuals with lymphoma (*r* = 0.93), suggesting that there is a good correlation between antibody level and neutralizing activity in patients with lymphoid malignancies^18^.

### Responses to third dose is defined by timing of treatment

**Fig. 6:**
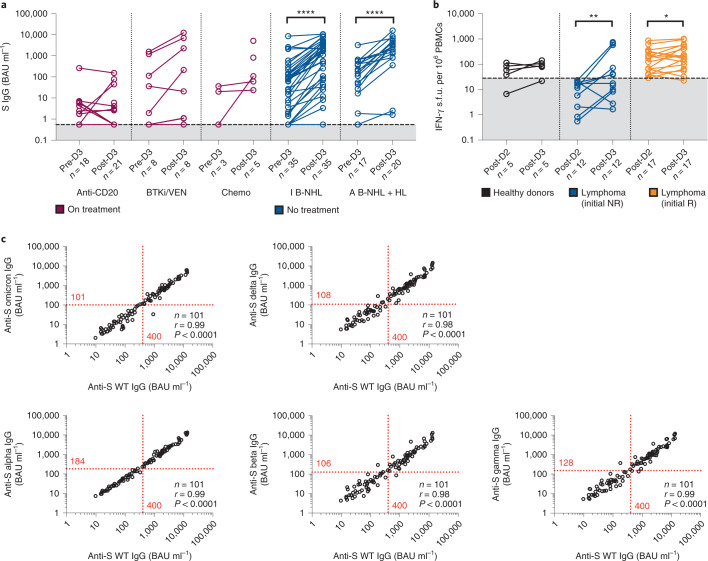
Antibody and pseudoneutralization responses to third vaccine dose. **a**, Antibody responses to the third vaccine dose according to therapy; concurrent or anti-CD20 therapy within 6 months in B-NHL (anti-CD20), concurrent BTK inhibitors or venetoclax in B-NHL (BTKi/VEN) and concurrent chemotherapy in HL (chemo), compared to those not on treatment (I B-NHL and A B-NHL and HL); two-sided Wilcoxon test, ****P < 0.0001. **b**, IFN-γ T-cell responses in healthy donors and participants with lymphoma who had a negative result (initial NR) or positive result (initial R) after two vaccine doses; two-sided Wilcoxon test, *P = 0.0361, **P = 0.0122. **c**, Good agreement is observed between binding of participant IgG to the wild-type SARS-CoV-2 and Omicron, Delta, Alpha, Beta and Gamma variants, undertaken in pre-D3 and post-D3 samples. The antibody concentration for each variant (value on inside of the *y* axis) equivalent to anti-S wild-type IgG 400 BAU ml^−1^ is shown; two-sided Spearman correlation and simple linear regression. Source data

**Extended Data Fig. 1:**
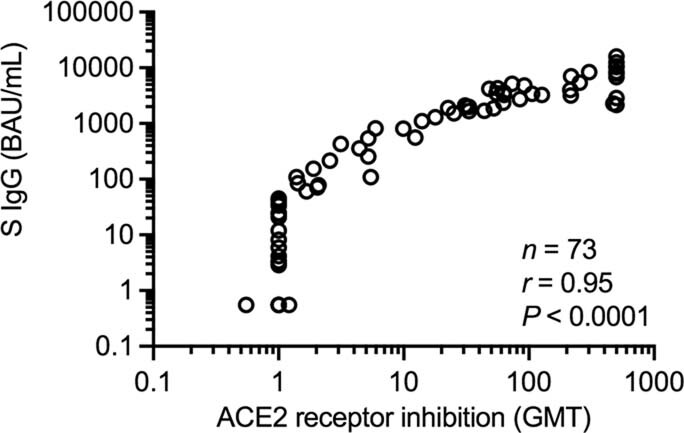
Correlation between anti-S IgG and ACE2 reception inhibition post third vaccine dose. Good correlation was observed between anti-S IgG antibody levels in post-D3 samples and ACE2 reception inhibition. Spearman correlation test, two-tailed. Source data

Sixty-three participants were sampled 20 to 26 weeks after the second vaccine dose (pre-D3) and 86 participants after receiving the third dose (post-D3) (Fig. 1 and Fig. 6a–c). Seventeen percent (3 out of 18) of participants who received their third vaccine dose within 52 weeks of anti-CD20 administration demonstrated a rise in anti-S level in contrast with 75% (6 out of 8) of participants with B-NHL who were on concurrent BTK inhibitor or venetoclax therapy and 100% (3 out of 3) of patients with HL who were on chemotherapy (Fig. 6a and Supplementary Table 4a). In participants who were treatment-naive or had completed systemic treatment more than 24 weeks before the third vaccine dose, the majority had improved antibody levels, including those with indolent B-NHL (pre-D3 versus post-D3, GMC 33.44 versus 487.4 BAU ml^−1^). Despite this, 29% (10 out of 35) participants still had anti-S IgG level of <106 BAU ml^−1^ (the lowest quartile for antibody levels achieved by healthy donors after two doses) after the third dose. Good correlation was observed between ACE2 receptor inhibition activity and post-D3 anti-S IgG levels (Extended Data Fig. 1).

Twenty-nine participants were also examined for T-cell responses at post-D3 and compared to post-D2 time point (Fig. 6b and Supplementary Table 4b). Here, 50% (6 out of 12) participants who did not have a detectable cellular response after the second dose demonstrate a positive response and 94% (16 out of 17) of those who had a positive response after the second dose had a sustained positive response.

### Binding to variants of concern can be predicted by binding to wild-type strain

Of primary concern is the ability of these anti-S IgG antibodies to bind to other SARS-CoV-2 variants of concern (VOCs). One hundred and one samples from pre-D3 and post-D3 time points were tested for binding to wild-type spike as well as Alpha, Beta, Delta, Gamma and Omicron VOCs (Fig. 6c). A close correlation was observed between anti-S wild-type IgG concentration to all VOCs tested. However, a reduction in binding antibodies was observed to all VOCs, with the greatest reduction observed in binding to the Omicron, Beta and Delta variants.

## Discussion

Our prospective cohort study evaluating antibody and cellular responses in 457 participants with lymphoid malignancies shows that the timing of SARS-CoV-2 vaccination in relation to treatment is the strongest predictor of antibody response. No antibodies were detected after two vaccine doses, in 52.3% of participants who were vaccinated within 24 weeks of completing their treatment compared to 8.7% who had not received any systemic treatment within 24 weeks. The impact of treatment timing persisted for the third dose. Other than timing of treatment in relation to vaccination, the best predictors of antibody response were the peripheral blood B-cell count and serum immunoglobulin levels, consistent with data reported in autoimmune rheumatic disease^22^.

Anti-CD20 therapy markedly impaired antibody responses for at least 6 months after treatment. However, the preservation of antigen-specific T-cell responses may justify the continued vaccination of these patients, particularly if they are on a prolonged course of rituximab therapy. Other B-cell-directed therapies such as BTK inhibitors, ibrutinib and acalabrutinib, also impaired antibody responses but not T-cell responses in our cohort. However, the numbers of patients were too small to reach any definitive conclusion. Other studies have also reported antibody impairment with these therapies^10,11^.

Chemotherapy administered without anti-CD20, also impairs antibody responses, but to a far lesser extent. This is reflected by only 11% (3 out of 27) of chemotherapy recipients having undetectable antibodies compared to 60% (58 out of 96) in those receiving anti-CD20. However, the caveat being that this population largely consisted of patients with HL who tend to be younger than those with B-NHL. The majority of participants with other tumor types treated with chemotherapy also tend to mount antibody responses to the vaccine^8,23^.

Data on cellular responses post SARS-CoV-2 vaccination are limited and where reported, most cohorts tend to be relatively small or heterogeneous in disease type, with the exception of Ehmsen and colleagues who analyzed 323 participants, mostly comprising CLL and multiple myeloma^13,24,25^. There is an increasing body of data demonstrating that T cells can protect individuals with impaired antibody responses to COVID-19 or after vaccination, against severe infection^2,26^. Consistent with other studies, we detected T-cell responses in participants on anti-CD20 (ref. ^25^). Due to the size of our dataset, we were also able to ascertain that systemic therapy had minimal effect on T-cell responses. One notable finding is the observation of anti-S T-cell responses in patients with HL regardless of treatment. It has long been suggested that these patients have a defect in their cellular immunity but our data show no evidence of excess functional T-cell impairment when compared to B-NHL^27,28^.

Reduced T-cell responses were observed in participants with indolent B-NHL who had not recently had systemic treatment, compared to aggressive B-NHL and HL. T-cell dysfunction has been previously reported in FL and CLL so this is not entirely unexpected^29,30^. Further, Wherry et al. also recently demonstrated elevated CD8^+^ T-cell responses in patients with multiple sclerosis treated with anti-CD20 (ref. ^26^). One of the mechanisms hypothesized was that anti-CD20 may deplete regulatory B cells, leading to loss of suppression of T cells. It could be that this phenomenon is apparent in indolent B-NHL and not aggressive B-NHL due to ‘lower baseline’ T-cell function. We also observed reduced cellular responses in participants treated with bendamustine, raising the possibility that participants treated with bendamustine and rituximab, a combination frequently employed in indolent B-NHL, may result in impairment of both antibody and cellular responses and consequently, no protection against COVID-19. Unfortunately, the cohort is too small to draw any firm conclusions.

Our study has several limitations, first is that we did not assess the neutralizing efficacy of the antibodies against SARS-CoV-2 VOCs. However, these data can be extrapolated from our accompanying publication, which reports the function of these antibodies, measured in the same laboratory, against the variants^18^. The good agreement between antibody level and ACE2 receptor blocking against the wild-type spike protein, suggests that these patients have functionally similar antibodies to healthy volunteers. The second limitation is the observational nature of the study, which has meant that we have not had the opportunity to obtain a blood sample for all patients at all time points, due to the rapidity of the national vaccination program in the United Kingdom. Nevertheless, as far as we are aware, this is the largest cohort of patients with mature B-cell HL and B-NHL for which antibody, cellular and pseudoneutralization data are available. As vaccine immune responses are heterogeneous, a large sample size is necessary to control for potentially confounding factors.

The timing between vaccination and treatment completion continues to be a critical factor even with a third vaccine dose. Eighty-three percent of participants who received their third dose within 52 weeks after anti-CD20 exposure had no increase in antibody response. In contrast, those on other treatments such as chemotherapy, BTK inhibitors and venetoclax responded to a third dose. Notably, 94% of individuals with indolent B-NHL had higher antibody levels after the third dose, albeit a third still had antibody levels lower than 106 BAU ml^−1^, the lowest quartile value of anti-S IgG achieved by healthy participants in our study after two doses. This suggests that these individuals are likely to benefit from further vaccine doses.

In summary, we have demonstrated that the strongest predictor of antibody vaccine response in a large cohort of participants with lymphoid malignancies is the timing of treatment in relation to vaccination, regardless of the number of doses administered. These patients’ antibodies have the same functional ability as healthy participants to block viral spike protein binding to the host ACE2 receptor and vaccine cellular responses are preserved in most. Anti-S IgG levels to VOCs are highly correlated with and thus can be extrapolated from wild-type levels, potentially enabling an antibody-based correlate of protection to be established across VOCs. Individuals with indolent B-NHL who have impaired antibody responses regardless of treatment, may benefit from antibody monitoring to identify those with suboptimal responses and thus could benefit from further vaccine doses. Finally, those who receive B-cell-depleting treatments such as anti-CD20, should be re-vaccinated 6 to 12 months after treatment completion.

## Methods

### Experimental model and participant details

#### Study design and patient data collection

This study was approved by the UK National Health Service Health Research Authority (North West-Liverpool Central Research Ethics Committee, IRAS 294739; 233768). It is a multicenter, prospective observational cohort study with the aim to investigate the immune responses from SARS-CoV-2 vaccination in individuals with lymphoid malignancy. The primary objective was to evaluate the robustness and persistence of COVID-19 vaccine immune responses in all individuals within 12 months of administration through evaluation of anti-S IgG antibodies. The secondary objectives were to identify baseline clinical parameters associated with reduced COVID-19 immune responses between four predetermined group of lymphoid cancers, the impact of no/previous treatment versus active treatment and the influence of the type of treatment received, by comparing anti-S IgG levels. Further, the incidence of symptomatic, virologically proven COVID-19 in all vaccinated individuals within 12 months of administration was assessed through capturing positive SARS-CoV-2 PCR results.

Participants were enrolled from local hospital databases or outpatient clinics from 11 March to 10 September 2021, after informed consent. No monetary compensation was provided. Eligible participants had to be 18 years old or older and have a confirmed diagnosis of a mature lymphoid malignancy. Information regarding demographic details, height and weight, disease characteristics, cancer treatment history, comorbidities, COVID-19 infection status, medication history, SARS-CoV-2 vaccination details and adverse events were collected. Participants also underwent peripheral blood sampling before vaccination, 4 weeks after first dose, 2–4 weeks after second dose, either 24 weeks after second dose or 6 weeks before the third dose, and 4–8 weeks after the third dose.

Participants were recruited into four main diagnostic categories, HL, aggressive B-NHL, indolent B-NHL and PTCL based on their histological diagnosis, where available. Participants with NLPHL were classified as indolent B-NHL for the purpose of this study due to the treatment of this disease with anti-CD20 monoclonal antibodies. Participants were allocated into ‘no treatment’ versus ‘on treatment’ groups. Participants who had not received systemic anti-lymphoma therapy for their disease, or whose treatment completed 24 weeks before the first vaccine dose, were allocated to the ‘no treatment’ group. Participants in the ‘on treatment’ group either received their first vaccine dose within 24 weeks of completing systemic anti-lymphoma therapy or started systemic anti-lymphoma therapy within 4 weeks of the first vaccine dose. For uniformity, 1 month was defined as 4 weeks in this study.

Healthy controls were vaccinees who received a vaccine as part of the government rollout and donated serum after vaccination for essay evaluation through verbal consent.

### Anti-SARS-CoV-2 S, RBD and N IgG assay

Antibody quantification was undertaken using frozen serum or plasma in the World Health Organization (WHO) International Reference Laboratory for Pneumococcal Serology at University College London. Antibodies to SARS-CoV-2 antigens N, RBD and trimeric S antigen (wild-type Wuhan strain)^31^ as well as spike derived from the VOCs (Alpha, Beta, Gamma, Delta and Omicron) were measured using a qualified multiplex electrochemiluminescent assay (Meso Scale Discovery)^18^. Samples, a standard curve and quality control serum were diluted and run in duplicate on plates coated with the relevant antigens. Bound IgG was detected using an anti-IgG antibody with a chemiluminescent tag. The assay was calibrated with the WHO International reference serum (NIBSC 20/136) and results expressed as BAU ml^−1^. Anti-N IgG concentration of 0.64 BAU ml^−1^ or lower, anti-RBD IgG of 0.73 BAU ml^−1^ or lower and anti-S IgG level of 0.55 BAU ml^−1^ or lower were below the detection limit. Participants with an anti-N IgG level exceeding 6.60 BAU ml^−1^ were considered to have encountered previous SARS-CoV-2 and were excluded from primary analysis.

The equations used to derive VOC values from anti-S wild-type IgG, where X is the value for anti-S wild-type are as follows:

Anti-S Omicron, *Y* = 0.395 × X − 57.58

Anti-S Delta, *Y* = 0.7628 × X − 197.2

Anti-S Alpha, Y = 0.8851 × X − 169.8

Anti-S Beta, *Y* = 0.6273 × X − 144.8

Anti-S Gamma, *Y* = 1.085 × X − 306.2

### Pseudoneutralization (ACE2 receptor blocking) assay

Samples with detectable anti-S and anti-RBD IgG antibodies were evaluated by a pseudoneutralization assay using the Meso Scale Discovery platform in the WHO International Reference Laboratory for Pneumococcal Serology at University College London^31^. A standard curve was derived from doubling dilutions of an anti-S monoclonal antibody as well as control or test serum (1:10 dilution) that were added to a 96-well MULTI-SPOT plate coated with S or RBD antigen. After incubation, ACE2-conjugated MSD SULFO-TAG was added and the plates were read using a MESO SECTOR S600 reader. The amount of ACE2 receptor blocking was calculated from the standard curve for each unknown serum and expressed as a titer. Samples that were above the maximum for the standard curve were assigned a titer of 2.5 times the maximum (500).

### SARS-CoV-2 IFN-γ enzyme-linked immunospot assay

SARS-CoV-2-specific T-cell responses were assessed using frozen PBMCs in a standardized enzyme-linked immunospot (ELISpot) assay at the WISH laboratory (compliant with Good Clinical Practice). Briefly, ELISpot plates were coated with anti-human IFN-γ antibody overnight. The following day, thawed cells were resuspended at 4 × 10^6^ cells ml^−1^ in Roswell Park Memorial Institute (RPMI) medium supplemented with 10% human AB serum, sodium pyruvate and l-glutamine and rested for 2 to 4 h at 37 °C and 5% CO_2_. The cells (approximately 4 × 10^5^ per well; 1 × 10^5^ for phytohemagglutin (PHA) (Sigma) wells) were plated in triplicate wells and incubated alone or with 1 µg ml^−1^ SARS-CoV-2 PepMix peptide pools (JPT) (peptide spanning the spike glycoprotein provided as two separate peptide pools) or 5 µg ml^−1^ PHA for 18–20 h. After incubation, PBMCs and peptides were washed off and plates were incubated with biotinylated anti-human IFN-γ (7-B6-1, Mabtech) for 90 min at 37 °C and 5% CO_2_. The plates were then washed with PBS-Tween four times before incubation with streptavidin alkaline phosphatase (Mabtech) for 60 min at 37 °C and 5% CO_2_. After four further washes with PBS-Tween, alkaline phosphatase chromogenic substrate (Novex) was added, spots were allowed to develop for ≤10 min and plates were read on an ELISpot plate reader (Autoimmun Diagnostika). Spots were assessed using ELISpot v.6.0 software and results were reported as s.f.u. per 10^6^ PBMCs. The mean of triplicate unstimulated wells was subtracted from individual replicate stimulated wells (for both peptide pool 1 and 2). The corrected values for each peptide pool were then summed and the mean of the total peptide pool response was calculated. The test result was considered positive if it was >28.06 s.f.u. per 10^6^ PBMCs. This value represents the upper 95% CI limit of 32 pre-vaccination samples.

### Peripheral blood T, B and NK cell quantification

Enumeration of peripheral blood T, B and NK cells was undertaken using multicolor flow cytometry^32^. Thawed PBMCs were washed twice in RPMI and suspended in FACS buffer and fluorescent-conjugated antibodies were added for 30 min at 2–8 °C before two washes and collected on a FACSCanto II (BD Biosciences). Data were analyzed using Cytobank.

PBMCs were analyzed for B cells (anti-CD19-PE-Cy7 and anti-CD3-FITC), CD4^+^ T cells (anti-CD3-FITC and anti-CD4-BV510), CD8^+^ T cells (anti-CD3-FITC and anti-CD8-APC eF780) and NK cells (anti-CD3-FITC and anti-CD56-PE) and markers of activation (HLA-DR-PerCP-Cy5.5 and CD38 APC). Counting beads (BioLegend) were used as per manufacturer’s instructions for cell counting.

### Statistics and reproducibility

The study sample size was calculated based on a precision as determined by the 95% CI for a proportion. This was an observational study and so no randomization was undertaken. Patient characteristics were summarized using descriptive statistics appropriate to the distribution, for example mean and s.d. for data that are approximately normal, and median and lower and upper quartiles for skewed data. No formal tests for normality were carried out, as these are highly sensitive to sample size, but instead distributions were assessed graphically for skew. The association between more than two continuous variables were tested using a two-sided Kruskal–Wallis rank-sum test with Dunn’s correction for multiple comparison. Where only two groups existed, a two-sided Mann–Whitney test was used. For binary outcomes, a logistic regression model was used for both univariable and multivariable analyses and odds ratios were reported with 95% CIs. All *P* values <0.05 were considered statistically significant. These analyses were exploratory and no formal adjustment was made for multiple testing. All analyses were performed in Stata v.16.0, Microsoft Excel for Mac v.15.37 and GraphPad Prism v.9.

### Reporting Summary

Further information on research design is available in the Nature Research Reporting Summary linked to this article.

## Supplementary information

Supplementary Information Supplementary Fig. 1 and Supplementary Tables 1–4

Reporting Summary

## Data Availability

Source data for Figs. 2 (except 2e), 3, 4 (except 4e), 5 and 6 and Extended Data Fig. 1 have been provided as Source Data Files. All other data are not publicly available due to them containing information that could compromise research participant privacy/consent. De-identified data supporting the findings will be available on completion of the study on reasonable request to the corresponding author after approval by an independent review committee. Proposals may be submitted up to 24 months after completion of the study. Source data are provided with this paper.

## Source data

Source Data Fig. 2 Statistical Source Data.
Source Data Fig. 3 Statistical Source Data.
Source Data Fig. 4 Statistical Source Data.
Source Data Fig. 5 Statistical Source Data.
Source Data Fig. 6 Statistical Source Data.
Source Data Extended Data Fig. 1 Statistical Source Data.
