# Supplementary material for: Immune responses against SARS-CoV-2 variants after two and three doses of vaccine in B-cell malignancies: UK PROSECO study
Source: Nat Cancer. 2022 Mar 24;3(5):552–64. doi: 10.1038/s43018-022-00364-3 (PMC9135622; doi:10.1038/s43018-022-00364-3)
Supplement: Supplementary file 1 — Supplementary Fig. 1 and Supplementary Tables 1–4 [file 43018_2022_364_MOESM1_ESM.pdf]

nature portfolio

https://doi.org/10.1038/s43018-022-00364-3

Supplementary information

Immune responses against SARS-CoV-2 variants after two and three doses of vaccine in B-cell malignancies: UK PROSECO study

In the format provided by the authors and unedited

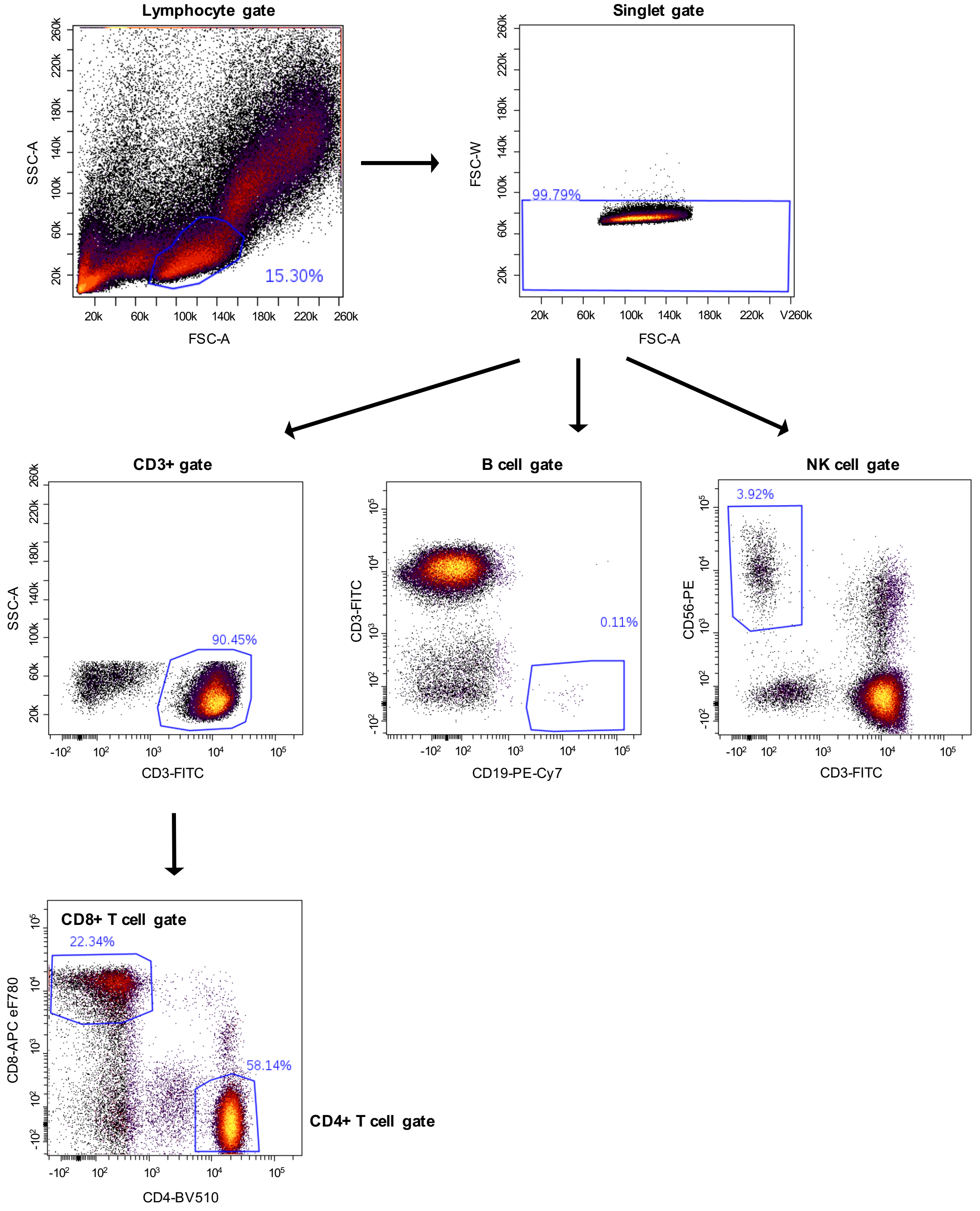

Supplementary Figure 1. Flow cytometry gating strategy.

The gating strategy used in this study for quantification of $CD4^{+}$ T, $CD8^{+}$ T, B and NK cell quantification.

	**OR favoring undetectable anti-S IgG (95% CI)**
**Treatment group**	
- **On**	**10.96 (6.44, 18.63; p<0.001)**
- **No**	Reference
**Vaccine**	
- **ChAdOx1**	Reference
- **BNT162b2**	0.96 (0.60, 1.52; p=0.854)
**Disease group**	
- **Aggressive BNHL**	Reference
- **HL**	**0.17 (0.05, 0.58; p=0.005)**
- **Indolent BNHL**	1.52 (0.93, 2.50; p=0.098)
- **Peripheral TNK**	1.59 (0.46, 5.52; p=0.464)
**Gender**	
- **Male**	Reference
- **Female**	0.93 (0.59, 1.47; p=0.758)
**Anti-CD20**	
- **No**	Reference
- **Yes**	**13.72 (7.78, 24.22; p<0.001)**
**Age**	**1.04 (1.02, 1.05; p<0.001)**
**Disease remission status**	
- **Complete/partial**	Reference
- **Stable disease**	0.77 (0.36, 1.67; p=0.513)
- **Progressive disease**	2.10 (0.76, 5.86; p=0.155)
**Serum IgG status**	
- **Normal/High**	Reference
- **Low**	**3.32 (1.62, 6.77 ; p=0.001)**

Supplementary Table 1. Univariable analysis of factors contributing to undetectable anti-S IgG. Univariable logistic regression reporting odds ratios and 95% confidence intervals. Older age, anti-CD20 treatment in the last 12 months, low serum IgG status and being in the 'on treatment' group were associated with higher risk of undetectable anti-S IgG.

	**OR favoring undetectable anti-S IgG (95% CI)**
**Treatment group**	
- **On**	**7.22 (3.80, 13.73; p<0.001)**
- **No**	Reference
**Vaccine**	
- **ChAdOx1**	Reference
- **BNT162b2**	0.89 (0.49, 1.62; p=0.695)
**Disease group**	
- **Aggressive B-NHL**	Reference
- **HL**	0.70 (0.16, 3.08; p=0.641)
- **Indolent B-NHL**	1.53 (0.80, 2.92; p=0.202)
- **PTCL**	2.60 (0.53, 12.76; p=0.240)
**Gender**	
- **Male**	Reference
- **Female**	0.82 (0.45, 1.50; p=0.525)
**Anti-CD20 administered in last 12 months**	
- **No**	Reference
- **Yes**	**5.60 (2.73, 11.52; p<0.001)**
**Age**	**1.03 (1.01, 1.06; p=0.011)**
**Disease remission status**	
- **Complete/partial**	Reference
- **Stable disease**	0.60 (0.26, 1.85; p=0.468)
- **Progressive disease**	2.44 (0.62, 9.51; p=0.200)

Supplementary Table 2. Multivariable analysis of factors contributing to undetectable anti-S IgG. Multivariable logistic regression model, reporting odds ratios and 95% confidence intervals. Older age, anti-CD20 treatment in the last 12 months and being in the 'on treatment' group were associated with higher risk of undetectable anti-S IgG.

	**OR favoring a positive IFNγ ELISpot response**
	OR (95% CI; p-value)
**Treatment group**	
- **On**	2.17 (0.99, 4.72; p=0.051)
- **No**	Reference
**Vaccine**	
- **ChAdOx1**	**2.01 (1.06, 3.79; p=0.031)**
- **BNT162b2**	Reference
**Disease group**	
- **Aggressive BNHL**	Reference
- **HL**	0.86 (0.26, 2.78; p=0.797)
- **Indolent BNHL**	0.64 (0.31, 1.34; p=0.238)
**Gender**	
- **Male**	Reference
- **Female**	0.67 (0.35, 1.28; p=0.230)
**Anti-CD20**	
- **No**	Reference
- **Yes**	0.47 (0.20, 1.08; p=0.076)
**Age**	1.00 (0.97, 1.02; p=0.774)
**Disease remission status**	
- **Complete/partial**	Reference
- **Stable disease**	1.13 (0.37, 3.40; p=0.834)
- **Progressive disease**	0.75 (0.18, 3.16; p=0.698)

Supplementary Table 3. Multivariable analysis of factors favoring reduced cellular responses. Multivariable logistic regression model, reporting odds ratios and 95% confidence intervals. ChAdOx1 vaccination vaccine was the only significant predictor of positive IFNγ ELISpot response.

a

	On Treatment			No Treatment	
	Anti-CD20	BTKi/VEN	Chemo	I B-NHL	A B-NHL & HL
Increased anti-S post-D3 relative to pre-D3, n (%)	3/18 (17%)	6/8 (75%)	3/3 (100%)	33/35 (94%)	16/17 (94%)

b

	Healthy Donors	Lymphoma	
		Initial Non-Responder	Initial Responder
Positive ELISpot at post-D3 relative to pre-D3, n (%)	4/5 (80%)	6/12 (50%)	16/17 (94%)

Extended Data Table 4. Antibody and cellular responses after third vaccine dose. a, The number and percentage of participants whose antibody levels increased after the third vaccine dose in each of the indicated groups (concurrent or anti-CD20 therapy within 6 months in B-NHL (anti-CD20), concurrent BTK inhibitors or venetoclax in B-NHL (BTKi/VEN) and concurrent chemotherapy in HL (chemo), compared to those not on treatment (I B-NHL and A B-NHL & HL). **b,** Number of participants who had positive IFNγ T-cell responses in healthy donors, and lymphoma participants after three doses in those who had a negative (initial non-responder) or positive (initial responder) after two vaccine doses.
