# Supplementary material for: Immune responses against SARS-CoV-2 variants after two and three doses of vaccine in B-cell malignancies: UK PROSECO study
Source: Nat Cancer. 2022 Mar 24;3(5):552–64. doi: 10.1038/s43018-022-00364-3 (PMC9135622; doi:10.1038/s43018-022-00364-3)
Supplement: Supplementary file 2 — Reporting Summary [file 43018_2022_364_MOESM2_ESM.pdf]

Corresponding author(s): Sean Hua Lim
Last updated by author(s): 2022-March-02

Reporting Summary

Nature Portfolio wishes to improve the reproducibility of the work that we publish. This form provides structure for consistency and transparency in reporting. For further information on Nature Portfolio policies, see our Editorial Policies and the Editorial Policy Checklist.

Statistics

For all statistical analyses, confirm that the following items are present in the figure legend, table legend, main text, or Methods section.

n/a	Confirmed	
☐	☒	The exact sample size (*n*) for each experimental group/condition, given as a discrete number and unit of measurement
☐	☒	A statement on whether measurements were taken from distinct samples or whether the same sample was measured repeatedly
☐	☒	The statistical test(s) used AND whether they are one- or two-sided<br>*Only common tests should be described solely by name; describe more complex techniques in the Methods section.*
☐	☒	A description of all covariates tested
☐	☒	A description of any assumptions or corrections, such as tests of normality and adjustment for multiple comparisons
☐	☒	A full description of the statistical parameters including central tendency (e.g. means) or other basic estimates (e.g. regression coefficient) AND variation (e.g. standard deviation) or associated estimates of uncertainty (e.g. confidence intervals)
☐	☒	For null hypothesis testing, the test statistic (e.g. *F*, *t*, *r*) with confidence intervals, effect sizes, degrees of freedom and *P* value noted<br>*Give P values as exact values whenever suitable.*
☒	☐	For Bayesian analysis, information on the choice of priors and Markov chain Monte Carlo settings
☒	☐	For hierarchical and complex designs, identification of the appropriate level for tests and full reporting of outcomes
☒	☐	Estimates of effect sizes (e.g. Cohen's *d*, Pearson's *r*), indicating how they were calculated

Our web collection on statistics for biologists contains articles on many of the points above.

Software and code

Policy information about availability of computer code

Data collection	Data was collected using ALEA electronic case report forms designed for the PROSECO study by the University of Southampton Clinical Informatics Research Unit.
Data analysis	Data and statistical analysis were done in Stata, version 16.0, Microsoft Excel for Mac, version 15.37, GraphPad Prism, version 9, ELISpot version 6.0, and Cytobank version 9.0.

For manuscripts utilizing custom algorithms or software that are central to the research but not yet described in published literature, software must be made available to editors and reviewers. We strongly encourage code deposition in a community repository (e.g. GitHub). See the Nature Portfolio guidelines for submitting code & software for further information.

Data

Policy information about availability of data

All manuscripts must include a data availability statement. This statement should provide the following information, where applicable:

- Accession codes, unique identifiers, or web links for publicly available datasets
- A description of any restrictions on data availability
- For clinical datasets or third party data, please ensure that the statement adheres to our policy

Source data for Fig. 2 (except 2E), 3, 4 (except 4E), 5, 6 and Extended Data Fig. 1 have been provided as Source Data Files. All other data are not publicly available due to them containing information that could compromise research participant privacy/consent. De-identified data supporting the findings of this study will be available on completion of the study on reasonable request to the corresponding author after approval by an independent review committee. Proposals may be submitted up to 24 months after completion of the study.

Field-specific reporting

Please select the one below that is the best fit for your research. If you are not sure, read the appropriate sections before making your selection.

☒ Life sciences ☐ Behavioural & social sciences ☐ Ecological, evolutionary & environmental sciences

For a reference copy of the document with all sections, see nature.com/documents/nr-reporting-summary-flat.pdf

Life sciences study design

All studies must disclose on these points even when the disclosure is negative.

Sample size	The sample size was calculated based on precision as determined by the 95% confidence interval for a proportion. Overall, across the 457 individuals, the primary outcomes can be estimated to a precision of at least +/- 4.6%. A total sample size of 457 individuals also allows for the effects of baseline parameters and treatments on the primary outcomes to be explored across the whole sample, and within each lymphoma subtype in subgroup analyses.
Data exclusions	None excluded.
Replication	Anti-SARS-CoV-2 S, RBD and N IgG assay: No replication was undertaken.<br><br>Pseudoneutralization assay: No replication was undertaken.<br><br>SARS-CoV-2 IFNgamma ELISpot assay: Cells were plated in triplicate wells for the ELISpot assay. Spots were assessed using ELISpot 6.0 software - count settings used were a size of 5 pixels and intensity of 12 brightness units. Any spots indicating large aggregates of artefacts were manually removed. Attempts at replication was successful.<br><br>Peripheral blood T, B and NK cell quantification: No replication was undertaken.
Randomization	Allocation to the disease groups was not randomized. This is an observational study. The intervention of interest in this study is SARS-CoV-2 vaccination. Participants were offered these vaccinations in line with the national vaccination program and it would not have been appropriate or possible to randomize this exposure.
Blinding	All laboratory assays were undertaken in a blinded fashion to remove bias in result reporting.

Reporting for specific materials, systems and methods

We require information from authors about some types of materials, experimental systems and methods used in many studies. Here, indicate whether each material, system or method listed is relevant to your study. If you are not sure if a list item applies to your research, read the appropriate section before selecting a response.

Materials & experimental systems

n/a	Involved in the study
☐	☒ Antibodies
☒	☐ Eukaryotic cell lines
☒	☐ Palaeontology and archaeology
☒	☐ Animals and other organisms
☐	☒ Human research participants
☐	☒ Clinical data
☒	☐ Dual use research of concern

Methods

n/a	Involved in the study
☒	☐ ChIP-seq
☐	☒ Flow cytometry
☒	☐ MRI-based neuroimaging

Antibodies

Antibodies used	CD56-PE, clone TULY56, eBioscience, CAT 12-0566-42, LOT 2280687; CD8-APC eF780, clone SK1, eBioscience, CAT 47-0087-42, LOT 2255548; HLA-DR-PerCP-Cy5.5, clone LN3, eBioscience, CAT 45-9956-42, LOT 2154879; CD19-PE-Cy7, clone HIB19, eBioscience, CAT 25-0199-42, LOT 2307386; CD3-FITC, clone SK7, eBioscience, CAT 11-0036-42, LOT 2007721; CD38 APC, clone HIT2, Biolegend, 303510, LOT B255162; CD4 BV510, clone SK3, Biolegend, CAT 344634, LOT B305870.
Validation	Antibodies chosen were based on manufacturer's validation and also from previous studies (Lim et al. RIVA-a phase IIa study of rituximab and varlilumab in relapsed or refractory B-cell malignancies: a study protocol for a randomized controlled trial. Trials, 2018, and Turaj et al, Augmentation of CD134 (OX40)-dependent NK anti-tumor activity is dependent on antibody cross-linking. Scientific Reports 2018).

Human research participants

Policy information about studies involving human research participants

Population characteristics	All patients aged 18 years or older AND had a confirmed lymphoma diagnosis were eligible. The age, gender, disease remission status and treatment history of the participants are described in Table 1 of the manuscript. For the healthy donor population, the median age was 45 years (IQR 34-47) and 67% were female.
Recruitment	The participants were recruited using existing local databases and during planned visits to the hospital. A potential selection bias of the study is that individuals who are frail and have poorer performance status may not be approached for the study due to the requirement for repeated blood sampling. This may bias the data towards fitter participants, and potentially underestimate the proportion of patients with reduced immune responses.
Ethics oversight	This study was approved by the UK National Health Service Research Authority (North West-Liverpool Central Research Ethics Committee, IRAS 294739; 233768). The samples from healthy vaccinees were donated as part of a service evaluation of vaccine roll out provided in the community so is not categorised as research. Verbal consent was given for assay evaluation.

Note that full information on the approval of the study protocol must also be provided in the manuscript.

Clinical data

Policy information about clinical studies

All manuscripts should comply with the ICMJE guidelines for publication of clinical research and a completed CONSORT checklist must be included with all submissions.

Clinical trial registration	ClinicalTrials.gov NCT4858568
Study protocol	The full study protocol is available on requesting from the corresponding author.
Data collection	Data from patients were recruited into the study from 11th March to 10th September 2021 from 9 hospitals in the United Kingdom. Data was collected for the period up to 10th January 2022.
Outcomes	The aim of the UK PROSECO study is to investigate the immune responses from SARS-CoV-2 vaccination in individuals with lymphoid malignancy. To achieve this, the following specific objectives were set.<br>Primary objective: To evaluate the robustness and persistence of COVID-19 vaccine immune responses in all individuals within 12 months of administration. This will be measured by evaluation of anti-spike IgG antibodies against SARS-CoV-2 after each vaccine dose.<br><br>Secondary objectives<br>1) To identify baseline clinical parameters associated with reduced COVID-19 immune responses<br>a) between four predetermined group of lymphoid cancers,<br>b) and within each group, the impact of no/previous treatment vs active treatment,<br>c) and within active treatment groups, the influence of the type of treatment received.<br>These endpoints will be measured by comparing anti-Spike IgG antibody levels within each group.<br>2) To assess the incidence of symptomatic, virologically proven COVID-19 in all vaccinated individuals within 12 months of administration. This will be measured through positive SARS-CoV-2 PCR results.

Flow Cytometry

Plots

Confirm that:

☒ The axis labels state the marker and fluorochrome used (e.g. CD4-FITC).

☒ The axis scales are clearly visible. Include numbers along axes only for bottom left plot of group (a 'group' is an analysis of identical markers).

☒ All plots are contour plots with outliers or pseudocolor plots.

☒ A numerical value for number of cells or percentage (with statistics) is provided.

Methodology

Sample preparation	Frozen PBMCs were thawed, washed twice in RPMI and resuspended to 2 million cells/ml and 100 ul of cells were added to each tube. An antibody master mix was prepared and 30 ul of antibody added to the PBMC tubes for 30 minutes at 2-8 degree Celcius in the dark. The samples were then washed twice with 2 ml of FACS buffer and resuspended in 200 ul of FACS buffer.
Instrument	FACSCanto II (BD Biosciences)
Software	Data was acquired of FACS Diva 6 and analyzed using Cytobank 9.0.
Cell population abundance	No cell-sorting was undertaken.

Gating strategy

Lymphocytes were gated based on FSC-A vs SSC-A. From this, singlets were gated in FSC-A vs FSC-W plot. Then, CD3+ cells were gated from CD3+ vs SSC-A, followed by CD4+ and CD8+ T cells from the CD4 vs CD8 plot. B cells were gated from the singlet population in a CD19 vs CD3 plot. NK cells were gated from the singlet population in a CD56 vs CD3 plot.

☒ Tick this box to confirm that a figure exemplifying the gating strategy is provided in the Supplementary Information.
